# Supplementary material for: Synthesis of Halopyrazole Matrine Derivatives and Their Insecticidal and Fungicidal Activities
Source: Molecules. 2022 Aug 5;27(15):4974. doi: 10.3390/molecules27154974 (PMC9370413; doi:10.3390/molecules27154974)
Supplement: Supplementary file 1 [file molecules-27-04974-s001.zip › molecules-1838919-supplementary.pdf]

SUPPLEMENTARY INFORMATION

Synthesis of Halopyrazole Matrine Derivatives and Their Insecticidal and Fungicidal Activities

Xingan Cheng [1†], Huiqing He [1,4,†], Fangyun Dong [1], Chunbao Charles Xu [3], Hanhui Zhang [1], Zhanmei Liu [1], Xiaojing Lv [1], Yuehua Wu [1], Xuhong Jiang [1,*] and Xiangjing Qin [2,*]

1 Institute of Natural Product Chemistry, College of Chemistry and Chemical Engineering/Key Laboratory of Green Prevention and Control on Fruits and Vegetables in South China, Ministry of Agriculture and Rural Affairs,/Institute of Plant Health, Zhongkai University of Agriculture and Engineering ,Guangzhou 510225, China; anzai_28@163.com (X.C.); dfy0429@live.cn (F.D.); zhanghanhui23@163.com (H.Z.); liuzhanm@21cn.com (Z.L.); xjinglv@zhku.edu.cn (X.L.); hxwyh2016@163.com (Y.W.)

2 CAS Key Laboratory of Tropical Marine Bio-Resources and Ecology, Guangdong Key Laboratory of Marine Materia Medica, South China Sea Institute of Oceanology, Chinese Academy of Sciences (CAS), Guangzhou 510301, China;

3 Department of Chemical and Biochemical Engineering, Western University, London, ON N6A5B9, Canada; cxu6@uwo.ca

4 Guangzhou Inspecting Testing and Certification Group Co. Ltd., Guangzhou 511447, China; huiqinghe1994@163.com

* Correspondence: jiangxh69@163.com (X.J.); xj2005qin@126.com (X.Q.)

† These authors contributed equally to this work.

Content

Synthesis scheme of matrine derivative compounds 1-7 1
High performance liquid chromatogram 1
Infrared spectroscopy 2
Mass spectrum 3
Mass spectrum 6
Degradability 17

Synthesis scheme of matrine derivative compounds 1-7

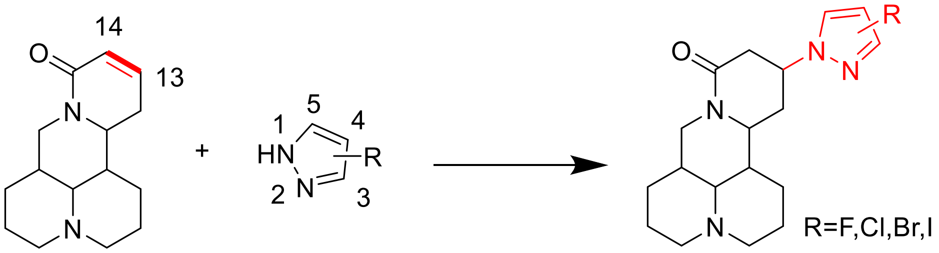

Figure S1 Synthesis scheme of matrine derivative compounds 1-7

Synthesis of compound 1 (3-chloropyrazole matrine derivative, 3-Cl-Pyr-Mat)

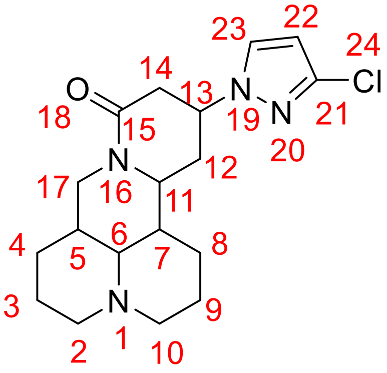

Sophocarpine (0.2460 g, 1.0 mmol), 3-chloropyrazole (0.1025 g, 1.0 mmol) and tripotassium orthophosphate (0.0500 g) were added into a 50 ml three-neck flask equipped with a condensation and reflux device. The reactants were dissolved in acetonitrile and stirred vigorously for 5 h at 90°C. Finally, a yellow-brown viscous liquid was obtained. The mixture was separated by column chromatography with ethyl ethanol: acetonitrile (1:10, v/v), and crystallized with ethyl acetate .

Data for compound 2. A colorless solid, 82.8% yield. HPLC (RT, min): 4.141. Selected IR data (KBr disk, cm^{-1}): υ (C-H, C=C) 3111 w; υ (C=O) 1645 s; υ (C-Cl) 755 m. ^{1}H NMR (700 MHz, Chloroform-d) δ 7.30 (d, J=2.41, 1H, H23), 6.16 (d, J=2.43, 1H, H22), 4.58 (dt, J=5.63, 2.73, 1H, H13), 4.33 (dt, J=12.73, 4.44, 1H, H11), 3.81 (d, J=5.39, 2H, H17), 3.03 (t, J=12.64, 1H, H6), 2.87 (d, J=6.00, 2H, H14), 2.81-2.72 (m, 4H, H2,10), 2.63-2.55 (m, 2H, H5,7), 2.15-1.84 (m, 2H, H12), 1.73-1.61 (m, 4H, H3,9), 1.58-1.21 (m, 4H, H4,8). ^{13}C NMR (176 MHz, Chloroform-d) δ 165.64 (C15), 139.69 (C21), 129.11 (C23), 105.35 (C22), 63.74 (C6), 57.22 (C11), 53.53 (C2,10), 50.33 (C13), 42.45 (C17), 42.02 (C14), 37.19 (C7), 35.69 (C12), 31.34 (C5), 27.75 (C4), 26.66 (C8), 21.18 (C9), 20.68 (C3). LRMS (ESI) m/z: calcd 348.17, observed 349.8 $[M+H]^{+}$.

Synthesis of compound 2 (3-bromopyrazole matrine derivative, 3-Br-Pyr-Mat)

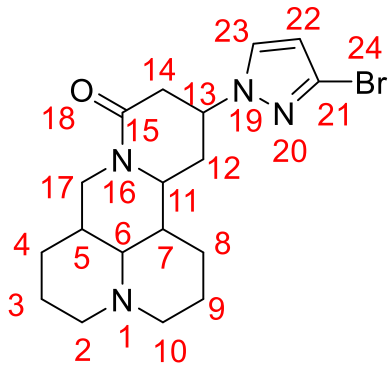

Sophocarpine (0.2460 g, 1.0 mmol), 3-bromopyrazole (0.1470 g, 1.0 mmol) and cesium carbonate (0.0500 g) were added into a 50 ml three-neck flask equipped with a condensation and reflux device. The reactants were dissolved in ultra-pure water and stirred vigorously for 8 h at 90°C. After a period of reaction, the mixture became clear light yellow. Finally, a yellow-brown viscous liquid was obtained. The mixture was separated by column chromatography with ethyl acetate: ethanol (16:1, v/v), and crystallized with acetonitrile.

Data for compound 3. A colorless solid, 84.5% yield. HPLC (RT, min): 4.356. Selected IR data (KBr disk, cm^{-1}): υ (C-H, C=C) 3115 w; υ (C=O) 1626 s; υ (C-Br) 755 m. ^{1}H NMR (700 MHz, Chloroform-d) δ 7.32 (d, J=2.49, 1H, H23), 6.27 (t, J=2.39, 1H, H22), 4.64 (dt, J=9.08, 3.09, 1H, H13), 4.34 (dt, J=12.68, 5.09, 1H, H11), 3.84 (d, J=5.85, 2H, H17), 3.05 (dd, J=12.83, 3.90, 1H, H6), 2.88 (d, J=5.44, 2H, H14), 2.84-2.74 (m, 4H, H2,10), 2.60 (dt, J=13.09, 6.29, 2H, H5,7), 1.96 (d, J=11.38, 2H, H12), 1.77-1.35 (m, 8H, H3,4,8,9). ^{13}C NMR (176 MHz, Chloroform-d) δ 165.52 (C15), 129.22 (C23), 125.84 (C21), 108.61 (C22), 63.57 (C6), 57.01 (C11), 53.37 (C2,10), 50.20 (C13), 42.11 (C17), 41.85 (C14), 37.15 (C7), 35.49 (C12), 31.09 (C5), 27.52 (C4), 26.44 (C8), 20.94 (C9), 20.42 (C3). LRMS (ESI) m/z: calcd 392.12, observed 393.6 $[M+H]^{+}$.

Synthesis of compound 3 (3-iodopyrazole matrine derivative, 3-I-Pyr-Mat)

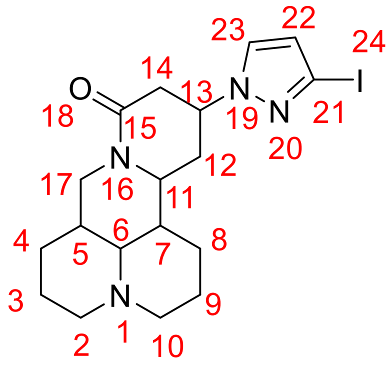

Sophocarpine (0.2460 g, 1.0 mmol), 3-iodopyrazole (0.1940 g, 1.0 mmol) and tripotassium orthophosphate (0.0300 g) were added into a 50 ml three-neck flask equipped with a condensation and reflux device. The reactants were dissolved in acetonitrile and stirred vigorously for 4 h at 90°C. A yellow-brown viscous liquid was finally obtained. The mixture was separated by column chromatography with ethyl acetate: ethanol (6:1, v/v), and crystallized with ethanol.

Data for compound 4. A colorless solid, 78.1% yield. HPLC (RT, min): 4.401. Selected IR data (KBr disk, cm^{-1}): υ (C-H, C=C) 3108 w; υ (C=O) 1624 s; υ (C-I) 759 m. ^{1}H NMR (700 MHz, Chloroform-d) δ 7.26 (d, J=2.40, 1H, H23), 6.41 (t, J=2.36, 1H, H22), 4.70 (tt, J=8.71, 3.05, 1H, H13), 4.33 (td, J=12.68, 4.45, 1H, H11), 3.83 (q, J=5.44, 2H, H17), 3.05 (t, J=12.63, 1H, H6), 2.88 (d, J=1.80, 2H, H14), 2.81-2.74 (m, 4H, H2,10), 2.60 (ddt, J=13.96, 8.34, 5.77, 1H, H7), 2.21-2.02 (m, 1H, H5), 1.98-1.86 (m, 2H, H12), 1.78-1.65 (m, 4H, H3,9), 1.62-1.36 (m, 4H, H4,8). ^{13}C NMR (176 MHz, Chloroform-d) δ 165.93 (C15), 129.55 (C23), 115.17 (C22), 95.15 (C21), 63.90 (C6), 57.36 (C11), 53.62 (C2,10), 50.55 (C13), 42.43 (C17), 42.20 (C14), 37.62 (C7), 35.83 (C12), 31.53 (C5), 27.87 (C4), 26.79 (C8), 21.28 (C9), 20.76 (C3). LRMS (ESI) m/z: calcd 440.11, observed 441.4 $[M+H]^{+}$.

Synthesis of compound 4 (4-fluoropyrazole matrine derivative, 4-F-Pyr-Mat)

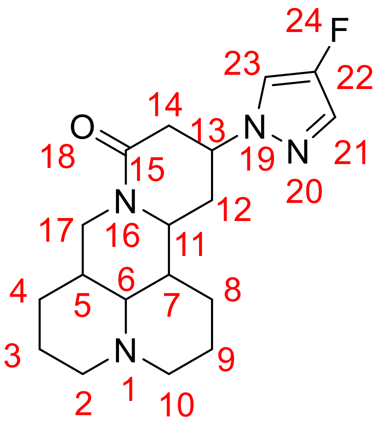

Sophocarpine (0.4920 g, 2.0 mmol), 4-fluoropyrazole (0.0860 g, 1.0 mmol) and tripotassium orthophosphate (0.0500 g) were added into a 50 ml three-neck flask equipped with a condensation and reflux device. The reactants were dissolved in 1,4-dioxane and stirred vigorously for 5.5 h at 105°C. A yellow-brown viscous liquid was finally obtained. The mixture was separated by column chromatography with ethanol: acetonitrile (1:5, v/v), and crystallized with ethyl acetate.

Data for compound 5. A colorless solid, 85.6% yield. HPLC (RT, min): 4.071. Selected IR data (KBr disk, cm^{-1}): ʋ (C-H, C=C) 3017 w; ʋ (C=O) 1634 s; ʋ (C-F) 813 m. ^{1}H NMR (500 MHz, Chloroform-d) δ 7.35 (s, 1H, H21), 7.34 (s, 1H, H23), 4.56 (dt, J=8.12, 2.80, 1H, H13), 4.35 (q, J=8.48, 1H, H11), 3.81 (d, J=4.57, 2H, H17), 3.05 (t, J=12.65, 1H, H6), 2.85 (m, 2H, H2,10), 2.77 (d, J=2.73, 2H, H14), 2.55-2.48 (m, 2H, H5,7), 2.14-2.00 (m, 2H, H2,10), 1.98-1.85 (m, 2H, H12), 1.75-1.64 (m, 4H, H4,8), 1.57-1.37 (m, 4H, H3,9). ^{13}C NMR (126 MHz, Chloroform-d) δ 165.20 (C15), 148.11 (C21), 125.98 (C23), 107.72 (C22), 63.12 (C6), 56.61 (C11), 53.06 (C2,10), 49.69 (C13), 41.86 (C17), 41.35 (C7), 36.63 (C14), 35.03 (C5), 30.80 (C12), 27.14 (C4) 26.05 (C8), 20.56 (C9), 20.05 (C3). LRMS (ESI) m/z: calcd 332.2, observed 333.8 $[M+H]^{+}$.

Synthesis of compound 5 (4-chloropyrazole matrine derivative, 4-Cl-Pyr-Mat)

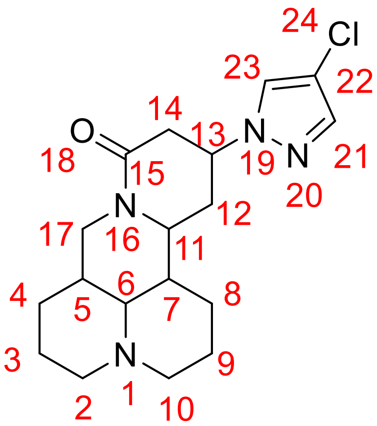

Sophocarpine (0.4920 g, 2.0 mmol), 4-chloropyrazole (0.1025 g, 1.0 mmol) and cesium carbonate (0.0500 g) were added into a 50 ml three-neck flask equipped with a condensation and reflux device. The reactants were dissolved in 1,4-dioxane and stirred vigorously for 7 h at 80°C. A yellow-brown viscous liquid was finally obtained. The mixture was separated by column chromatography with ethyl acetate/methanol (4:1, v/v), and crystallized with dichloromethane.

Data for compound 6. A colorless solid, 87.0% yield. HPLC (RT, min): 4.346. Selected IR data (KBr disk, cm^{-1}): υ (C-H, C=C) 31367 w; υ (C=O) 1624 s; υ (C-Cl) 838 m. ^{1}H NMR (500 MHz, Chloroform-d) δ 7.44 (s, 1H, H23), 7.41 (s, 1H, H21), 4.63 (dt, J=8.3, 2.8, 1H, H13), 4.36 (dq, J=12.7, 4.3, 1H, H11), 3.82 (d, J=5.3, 2H, H17), 3.061 (t, J=12.7, 1H, H6), 2.92-2.85 (m, 2H, H2,10), 2.78 (d, J=11.2, 2H, H14), 2.59-2.52 (m, 2H, H5,7), 2.17-2.01 (m, 2H, H2,10), 1.97-1.86 (m, 2H, H12), 1.75-1.64 (m, 4H, H4,8), 1.60-1.37 (m, 4H, H3,9). ^{13}C NMR (126 MHz, Chloroform-d) δ 164.98 (C15), 137.35 (C21), 124.60 (C23), 109.44 (C22), 62.93 (C6), 56.45 (C11), 56.41 (C10), 52.86 (C2), 49.51 (C13), 41.74 (C17), 41.19 (C7), 36.48 (C14), 34.88 (C5), 30.70 (C12), 26.99 (C4), 25.88 (C8), 20.41 (C9), 19.90 (C3). LRMS (ESI) m/z: calcd 348.17, observed 349.8 $[M+H]^{+}$.

Synthesis of compound 6 (4-bromopyrazole matrine derivative, 4-Br-Pyr-Mat)

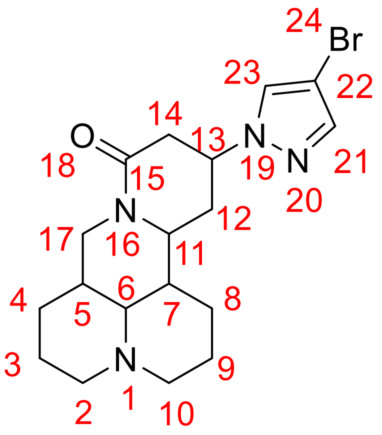

Sophocarpine (0.4920 g, 2.0 mmol), 4-bromopyrazole (0.2205 g, 1.5 mmol) and cesium carbonate (0.0500 g) were added into a 50 ml three-neck flask equipped with a condensation and reflux device. The reactants were dissolved in ethanol and stirred vigorously for 7 h at 90°C. A yellow-brown viscous liquid was finally obtained, was separated by column chromatography with ethyl acetate: ethanol (6:1, v/v), and crystallized with ethanol.

Data for compound 7. A colorless solid, 83.00% yield. HPLC (RT, min): 4.424. Selected IR data (KBr disk, cm^{-1}): υ (C-H, C=C) 3106 w; υ (C=O) 1636 s; υ (C-Br) 615 m. ^{1}H NMR (700 MHz, Chloroform-d) δ 7.47 (s, 1H, H23), 7.44 (s, 1H, H21), 4.65 (dt, J=8.35, 2.82, 1H, H13), 4.36 (dq, J=13.05, 3.24, 1H, H11), 3.84 (d, J=4.66, 2H, H17), 3.05 (t, J=12.93, 1H, H6), 2.97-2.84 (m, 2H, H2,10), 2.77 (d, J=12.22, 2H, H14), 2.59-2.53 (m, 2H, H5,7), 2.16-2.03 (m, 2H, H2,10), 1.98-1.87 (m, 2H, H12), 1.74-1.64 (m, 4H, H4,8), 1.59-1.39 (m, 4H, H3,9). ^{13}C NMR (176 MHz, Chloroform-d) δ 165.44 (C15), 139.83 (C21), 127.23 (C23), 93.01 (C22), 63.37 (C6), 56.81 (C11), 53.20 (C13), 49.95 (C2), 42.00 (C10), 41.59 (C17), 36.96 (C7), 35.26 (C14), 31.05 (C5), 29.43 (C12), 27.34 (C4), 26.22 (C8), 20.73 (C9), 20.22 (C3). LRMS (ESI) m/z: calcd 392.12, observed 393.6 $[M+H]^{+}$.

Synthesis of compound 7 (4-iodopyrazole matrine derivative, 4-I-Pyr-Mat)

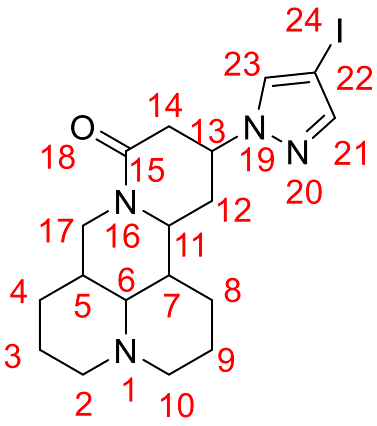

Sophocarpine (0.2460 g, 1.0 mmol), 4-iodopyrazole (0.1940 g, 1.0 mmol) and cesium carbonate (0.0500 g) were added into a 50 ml three-neck flask equipped with a condensation and reflux device. The reactants were dissolved in acetonitrile and stirred vigorously for 2.5 h at 90°C. Finally, a yellow-brown viscous liquid was resulted, followed by separation by column chromatography with ethyl acetate: ethanol (4:1, v/v) and crystallization in ethanol.

Data for compound 8. A colorless solid, 81.3% yield. Colorless solid. HPLC (RT, min): 4.539. Selected IR data (KBr disk, cm^{-1}): υ C-H, C=C) 3133 w; υ (C=O) 1623 s; υ (C-I) 622 m. ^{1}H NMR (700 MHz, Chloroform-d) δ 7.52 (s, 1H, H23), 7.45 (s, 1H, H21), 4.67 (dt, J=4.7, 4.1, 1H, H13), 4.36 (dq, J=12.73, 4.33, 1H, H11), 3.83 (d, J=5.57, 2H, H17), 3.05 (d, 1H, H6), 2.94-2.83 (m, 2H, H2,10), 2.79 (d, J=15.58, 2H, H14), 2.60-2.53 (m, 2H, H5,7), 2.18-2.00 (m, 2H, H2,10), 1.97-1.85 (m, 2H, H12), 1.76-1.47 (m, 4H, H4,8), 1.45-1.19 (m, 4H, H3,9). ^{13}C NMR (176 MHz, Chloroform-d) δ 165.52 (C15), 144.55 (C21), 131.48 (C23), 63.54 (C22), 57.00 (C11), 56.97 (C6), 53.24 (C13), 50.10 (C10), 42.25 (C2), 41.77 (C17), 37.16 (C7), 35.44 (C14), 31.29 (C9), 29.60 (C12), 27.53 (C4), 26.42 (C8), 20.94 (C3), 20.43(C9). LRMS (ESI) m/z: calcd 440.11, observed 441.40 $[M+H]^{+}$.

High performance liquid chromatogram

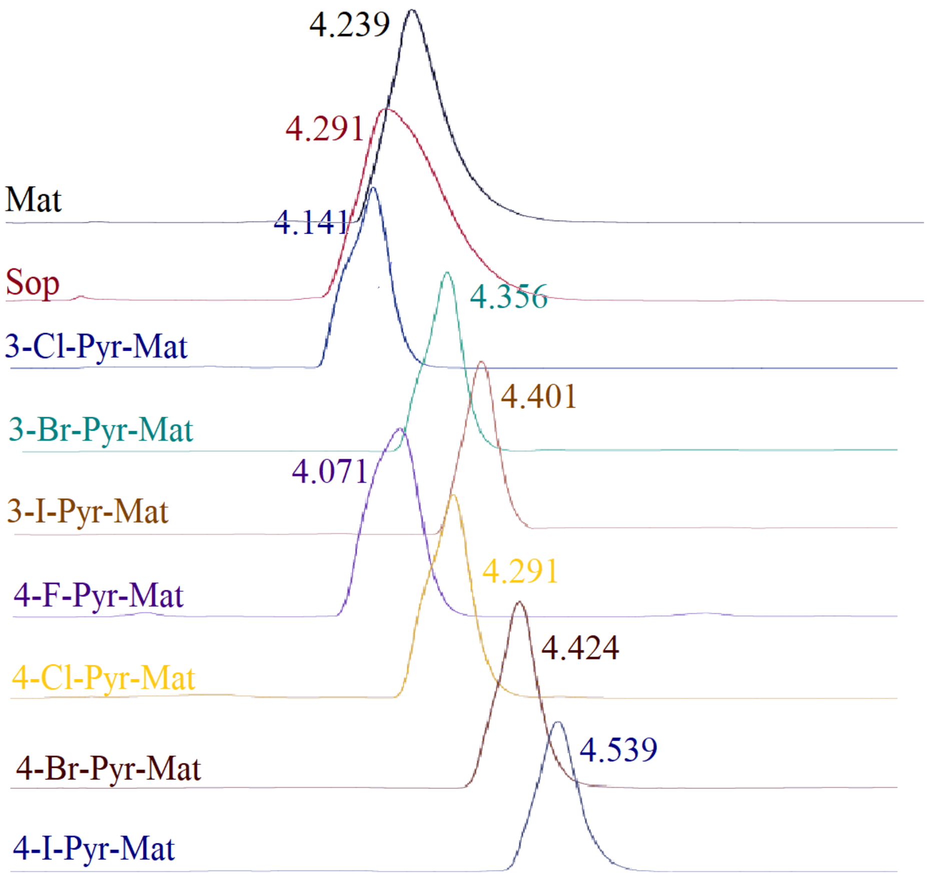

Figure S2　HPLC chromatograms of sophocarpine, matrine and compounds 1-7

Infrared spectroscopy

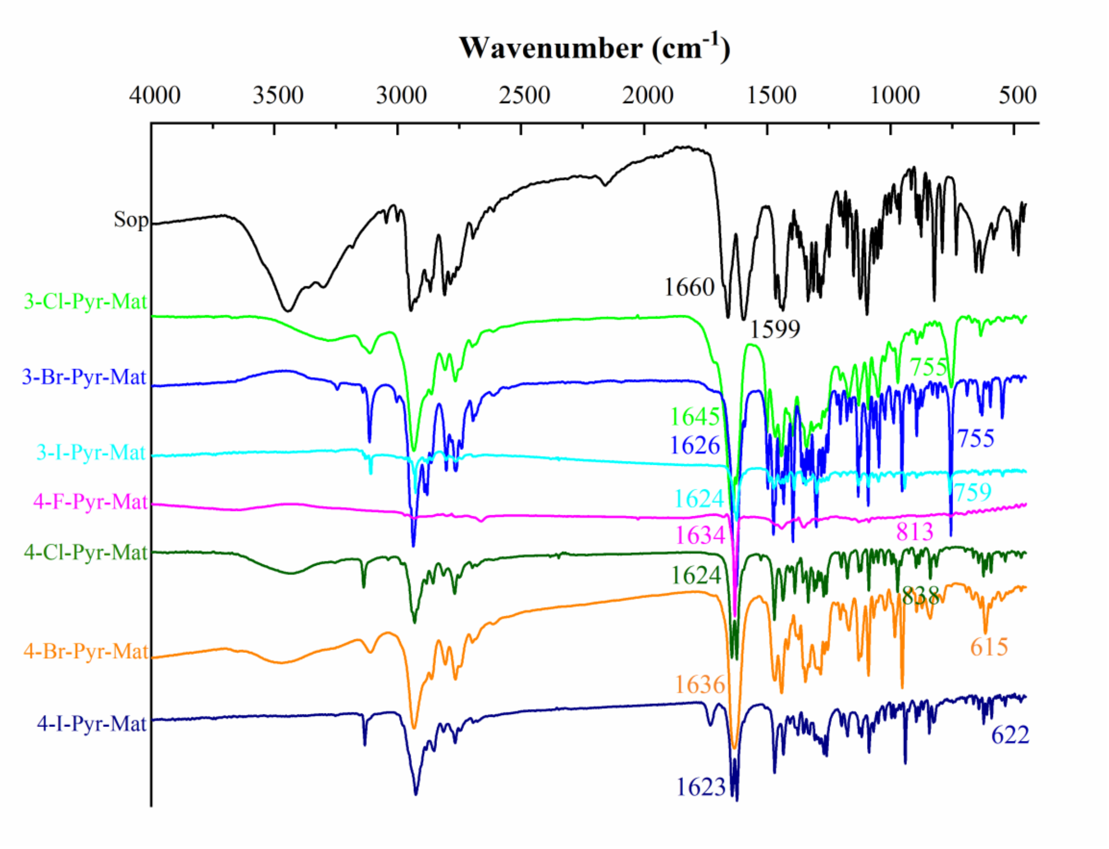

Figure S3 IR spectra of sophocarpine and compounds 1-7

Mass spectrum

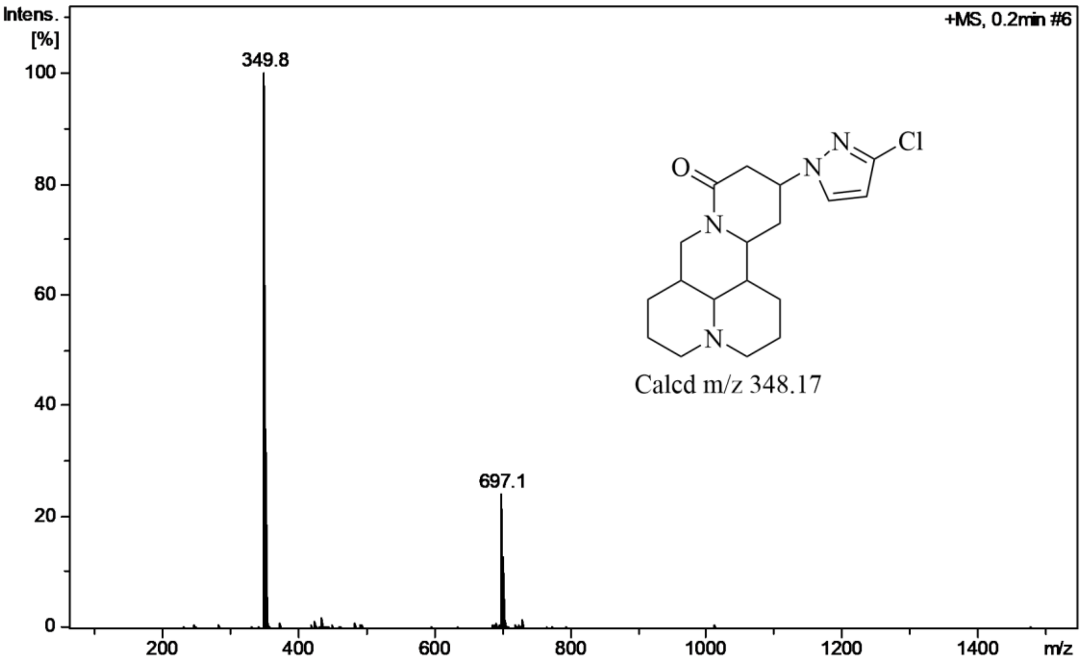

Figure S4 LR-ESI-MS of Compound 1

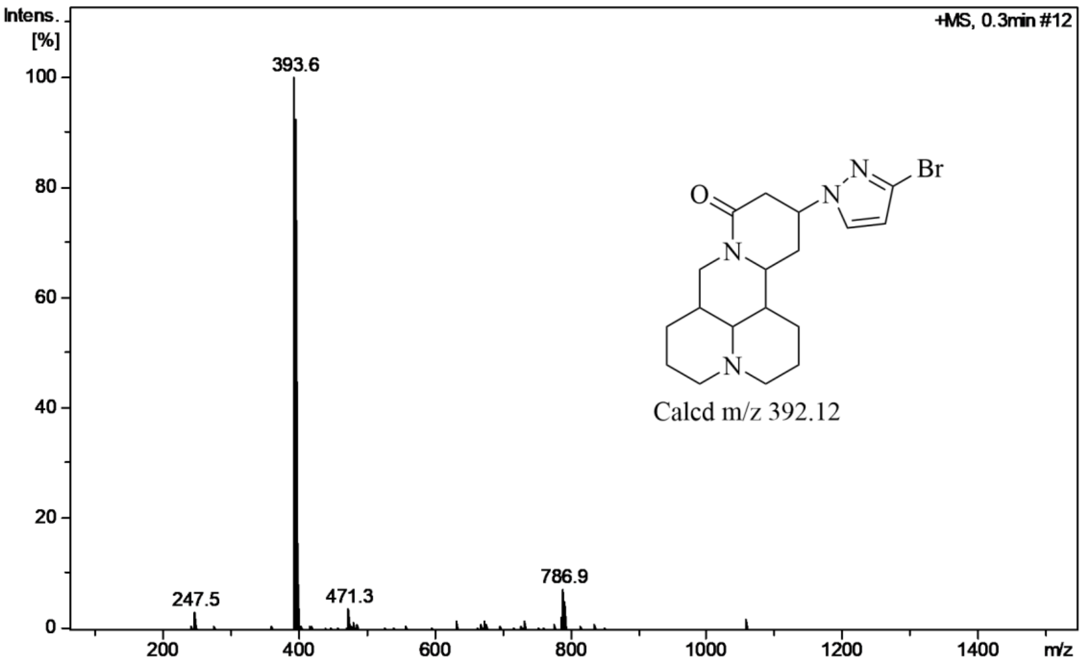

Figure S5 LR-ESI-MS of Compound 2

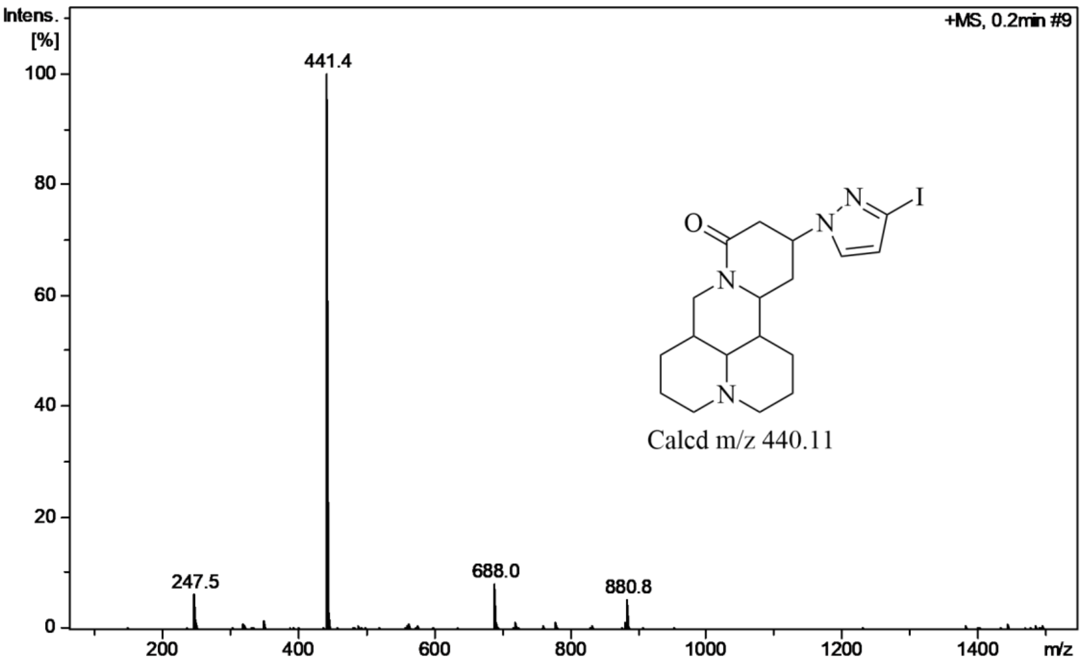

Figure S6 LR-ESI-MS of Compound 3

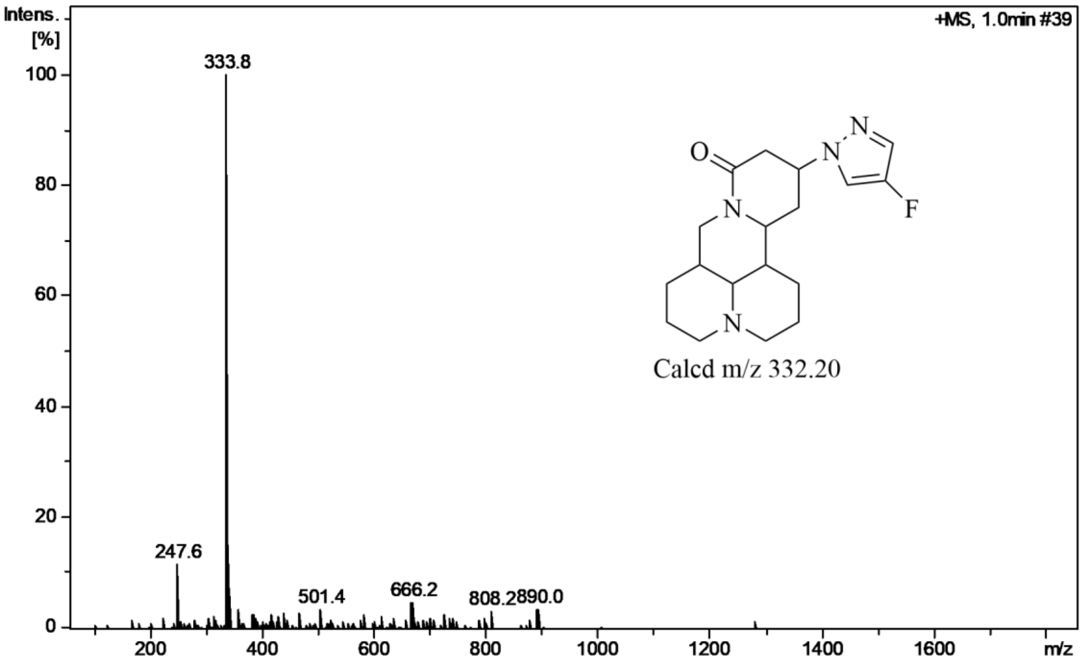

Figure S7 LR-ESI-MS of Compound 4

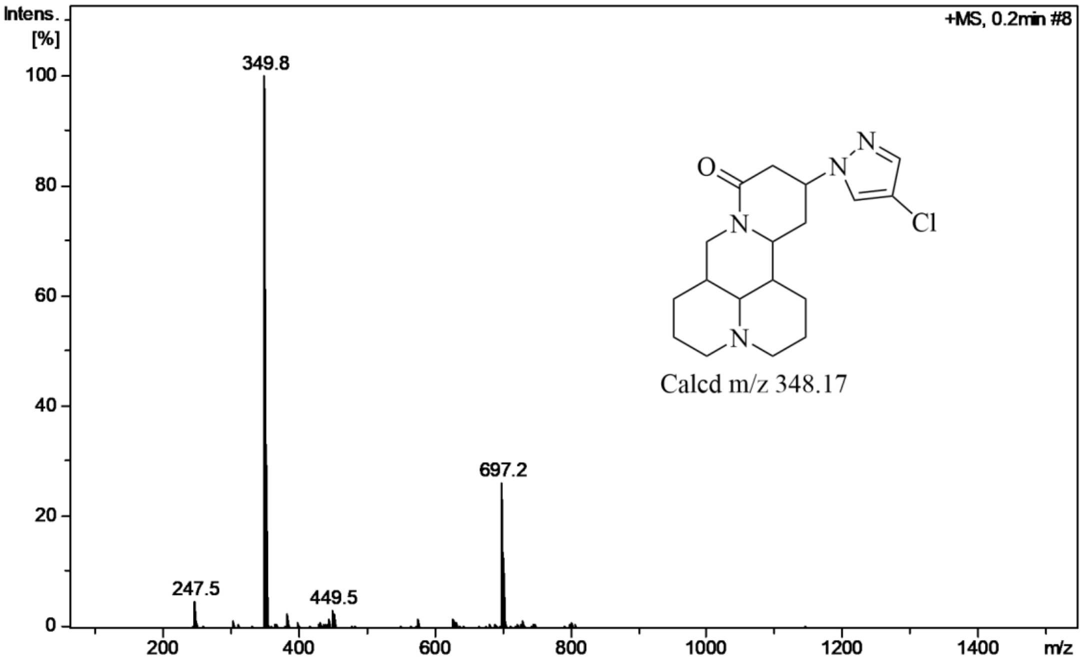

Figure S8 LR-ESI-MS of Compound 5

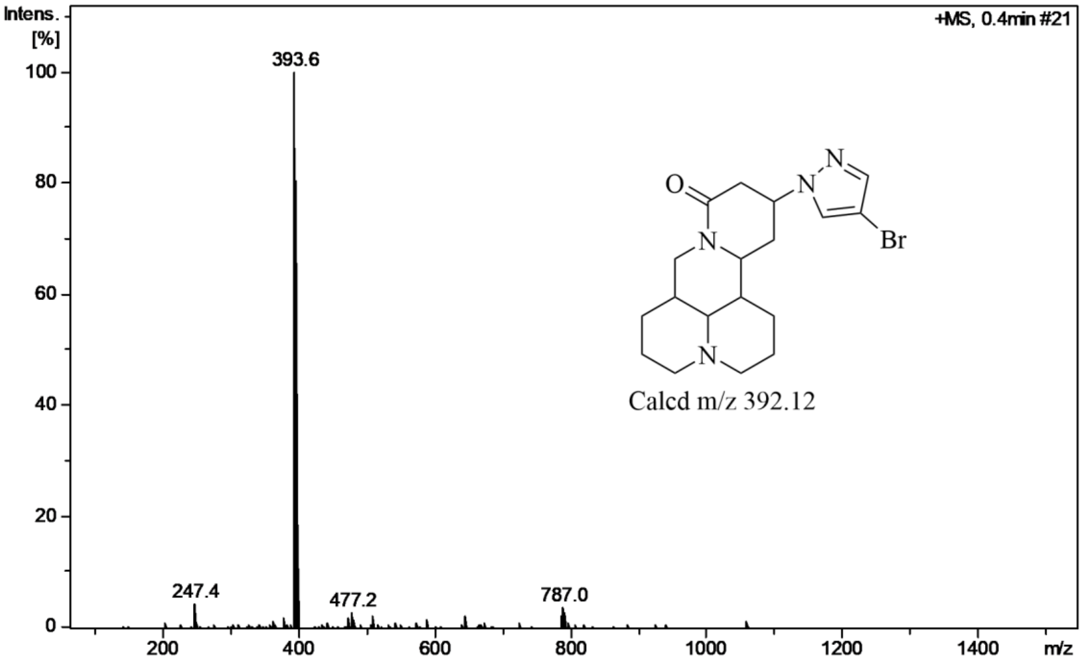

Figure S9 LR-ESI-MS of Compound 6

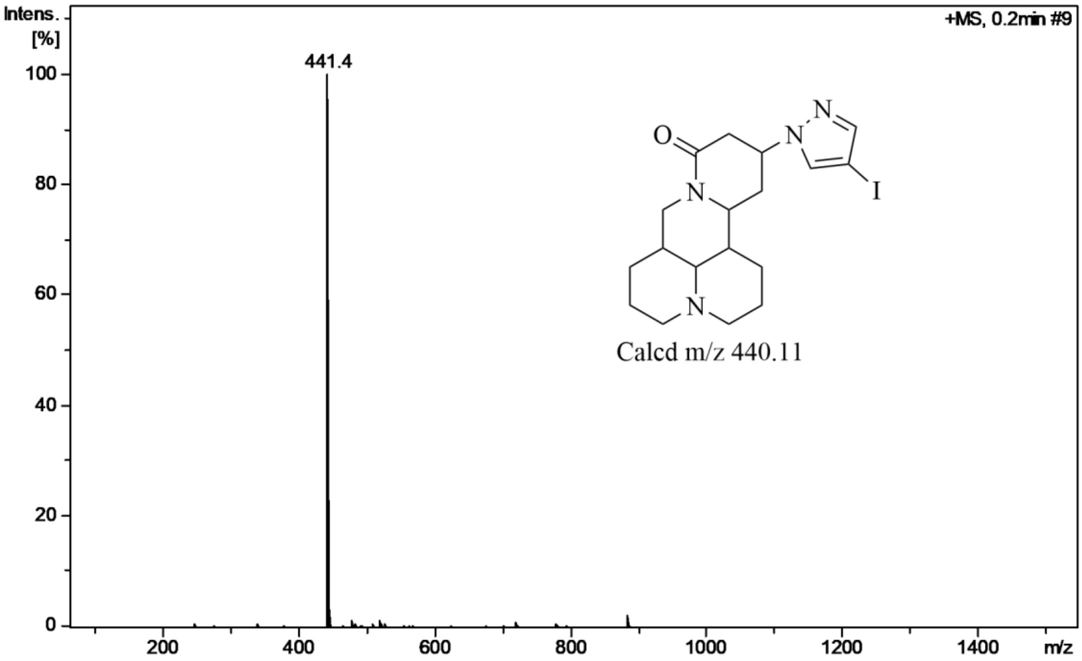

Figure S10 LR-ESI-MS of Compound 7

Mass spectrum

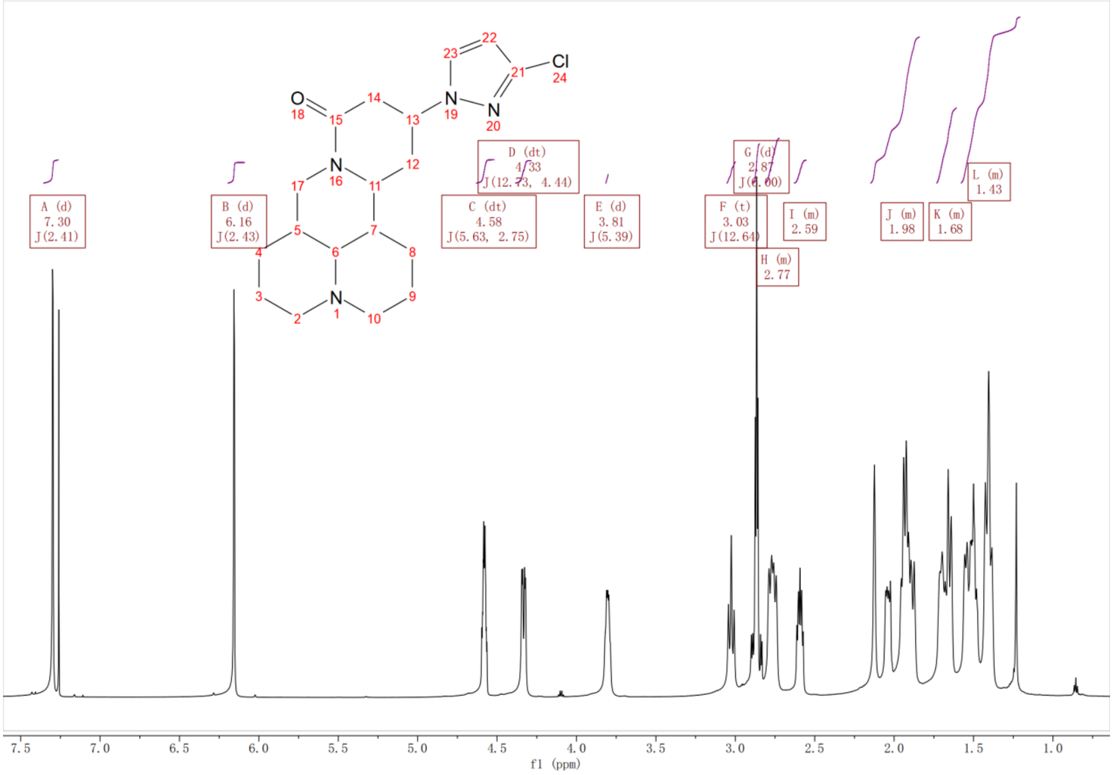

Figure S11 ^{1}H NMR spectrum of Compound 1

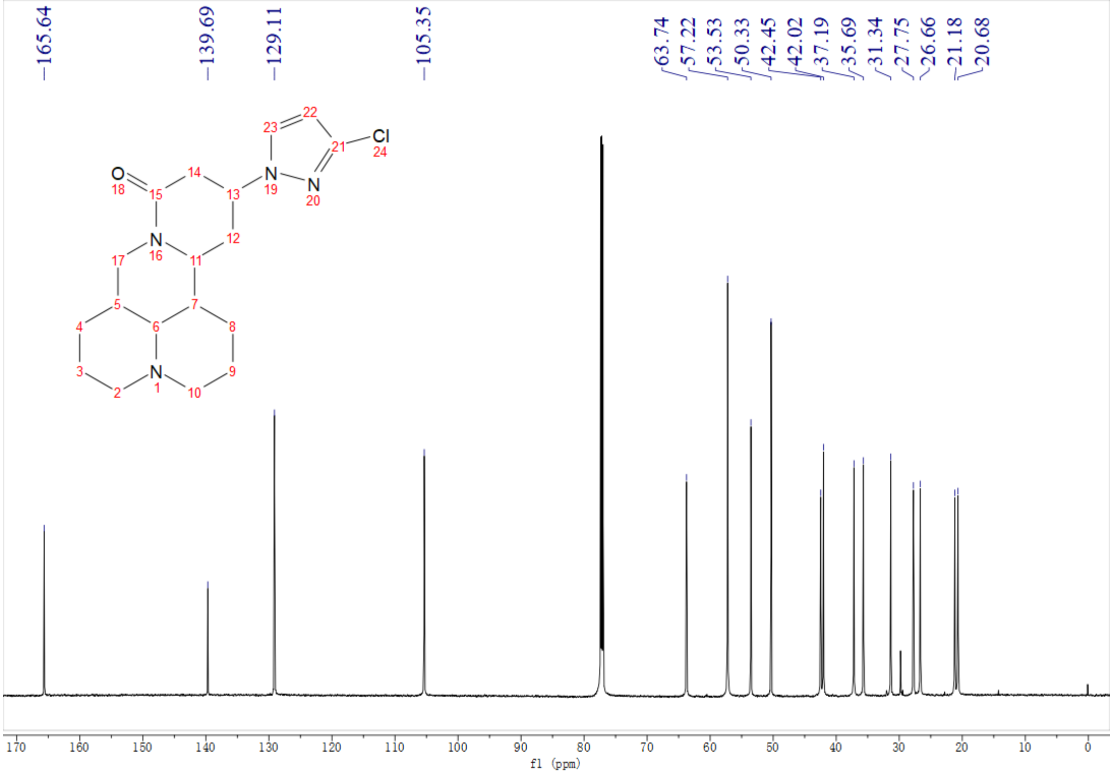

Figure S12 ^{13}C NMR spectrum of Compound 1

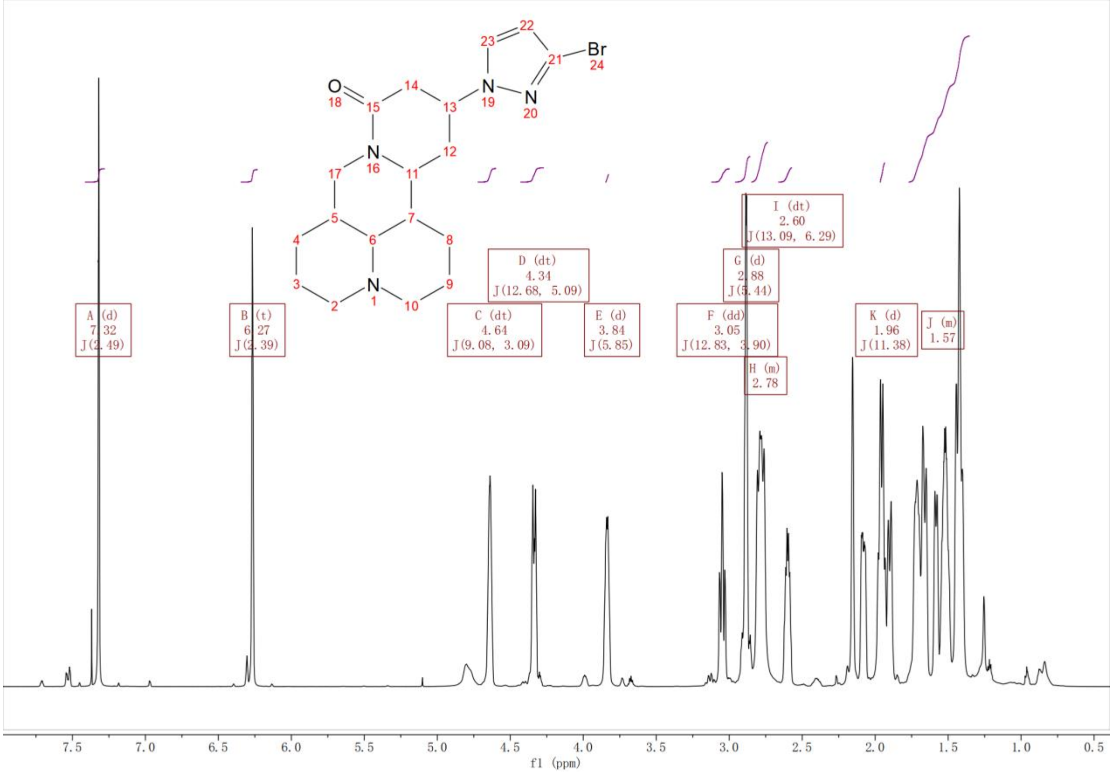

Figure S13 ^{1}H NMR spectrum of Compound 2

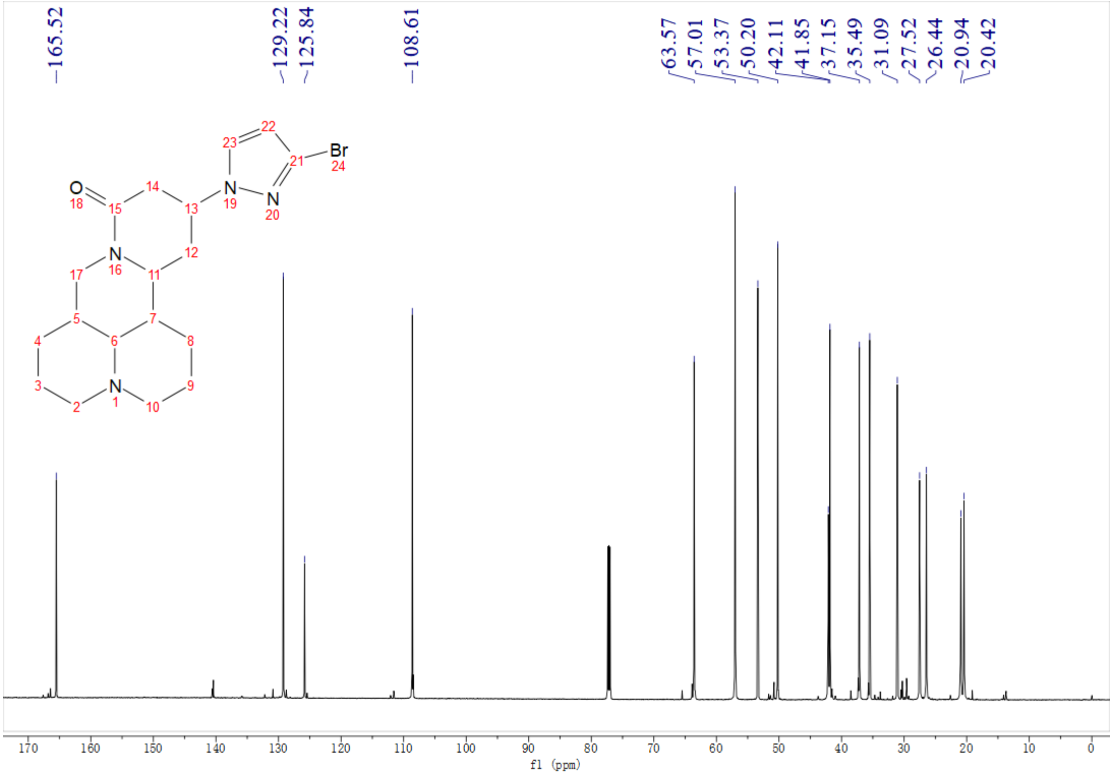

Figure S14 ^{13}C NMR spectrum of Compound 2

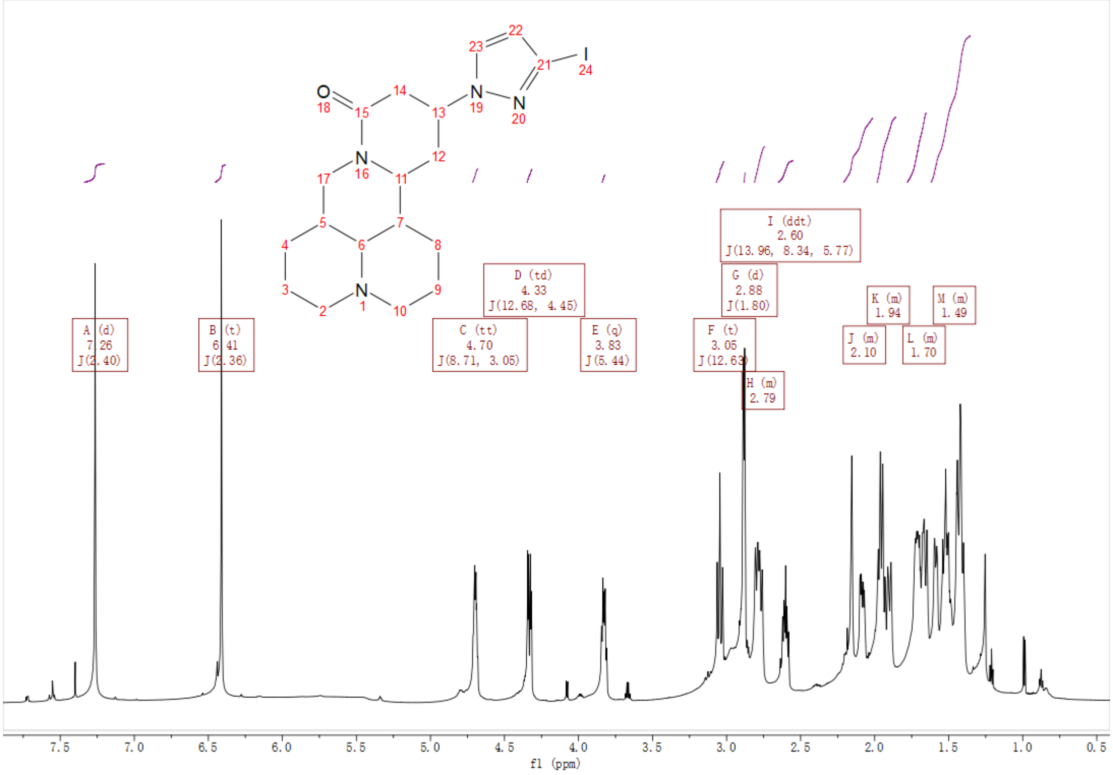

Figure S15 ^{1}H NMR spectrum of Compound 3

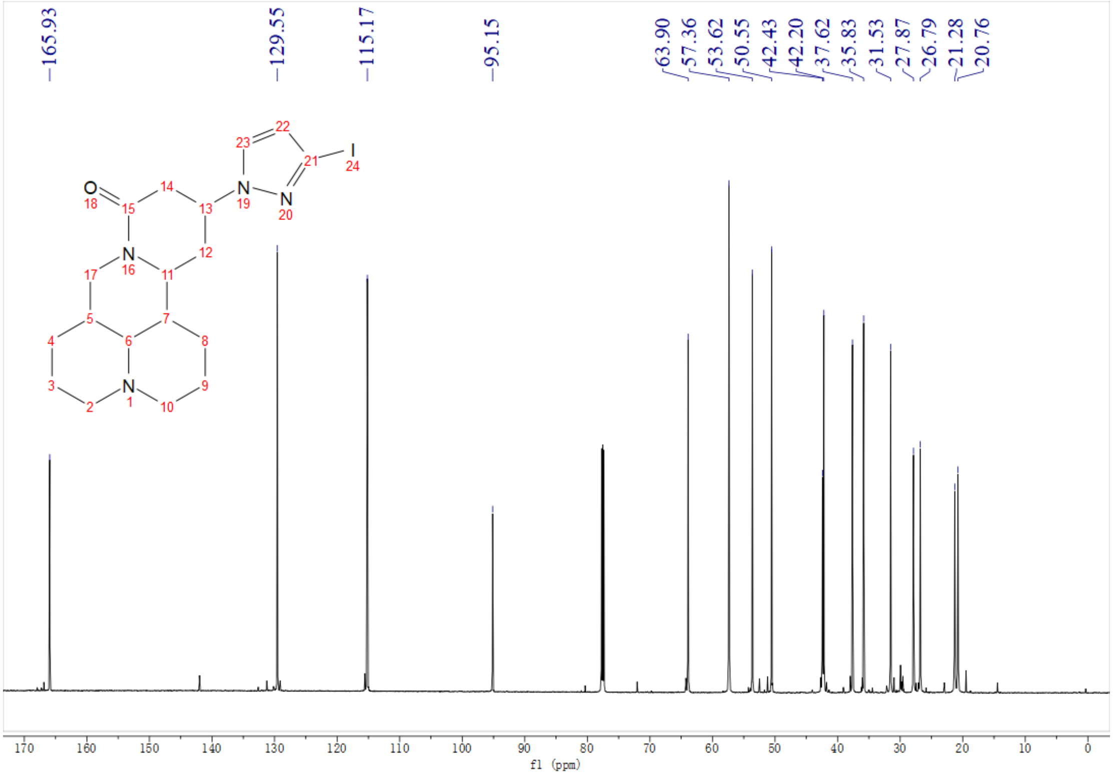

Figure S16 ^{13}C NMR spectrum of Compound 3

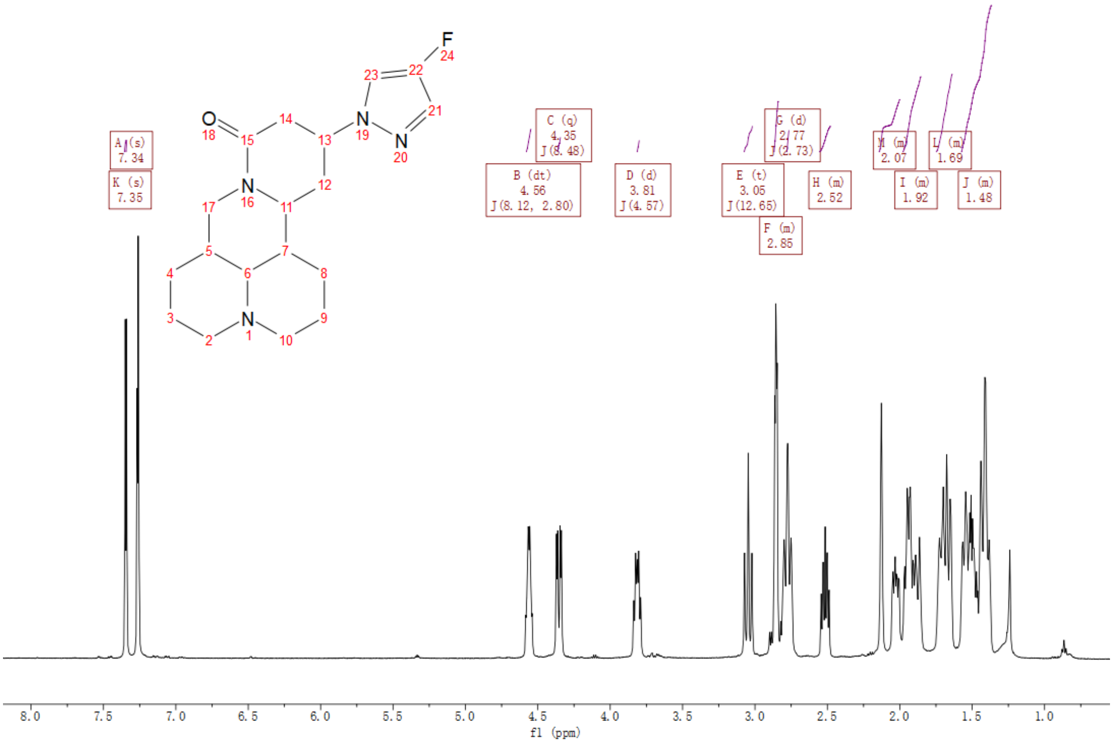

Figure S17 ^{1}H NMR spectrum of Compound 4

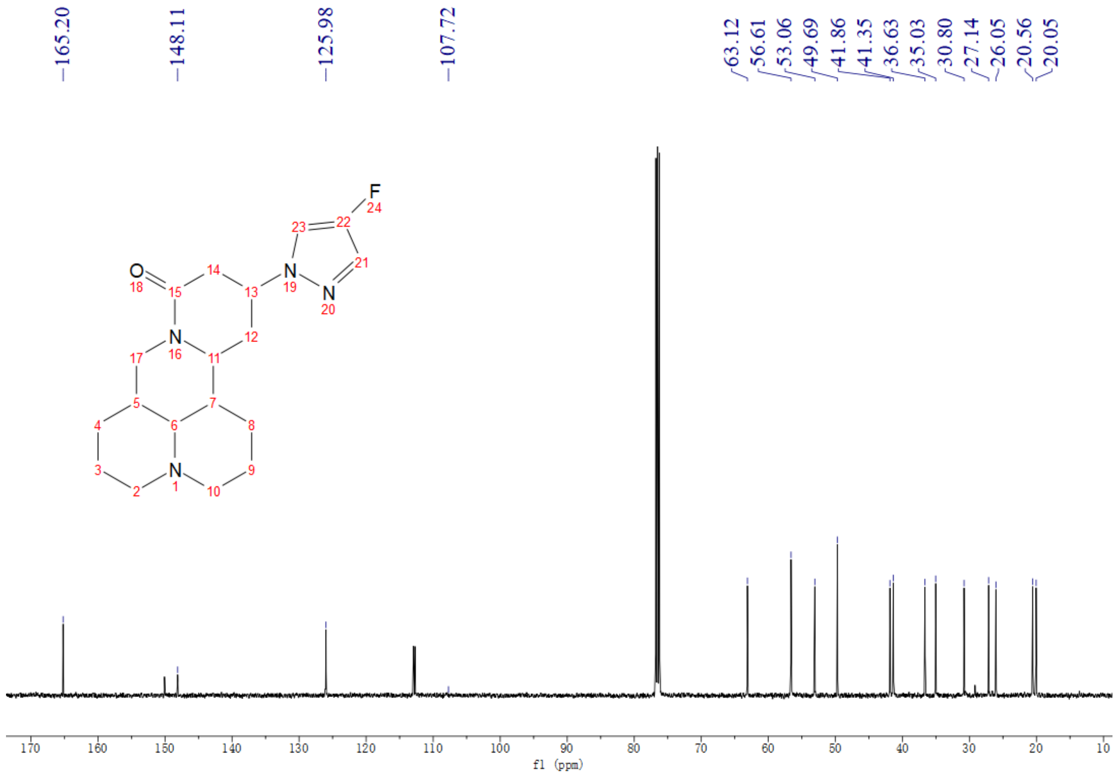

Figure S18 ^{13}C NMR spectrum of Compound 4

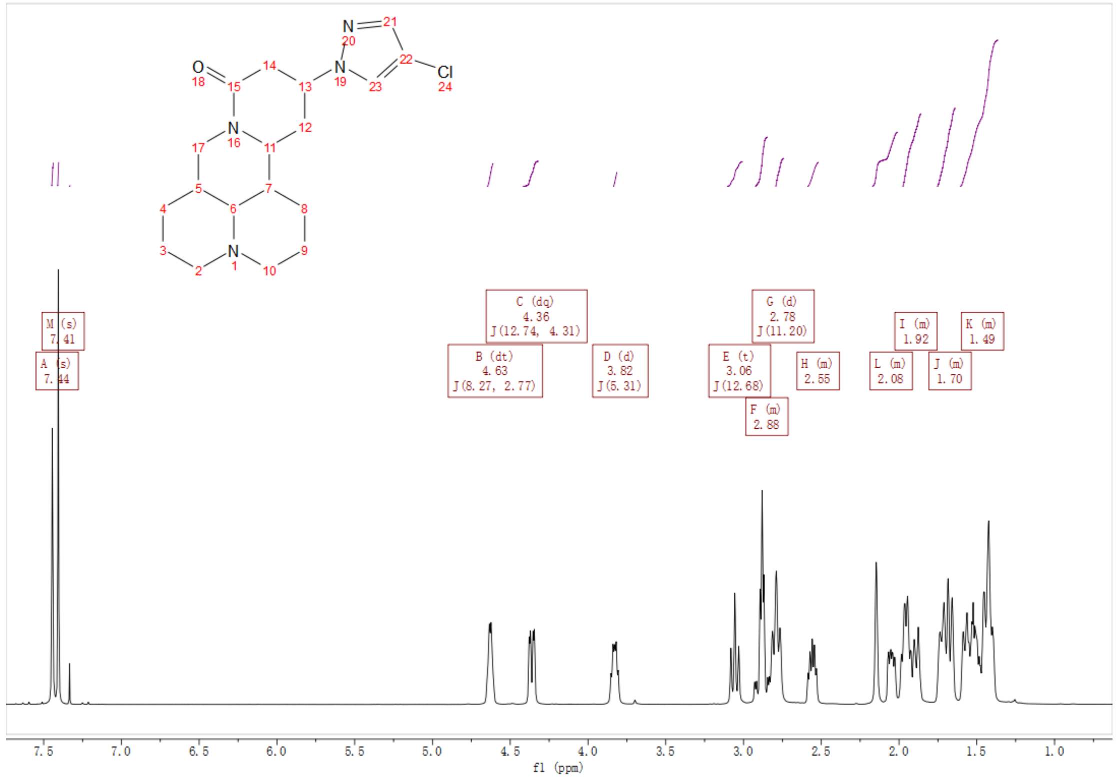

Figure S19 ^{1}H NMR spectrum of Compound 5

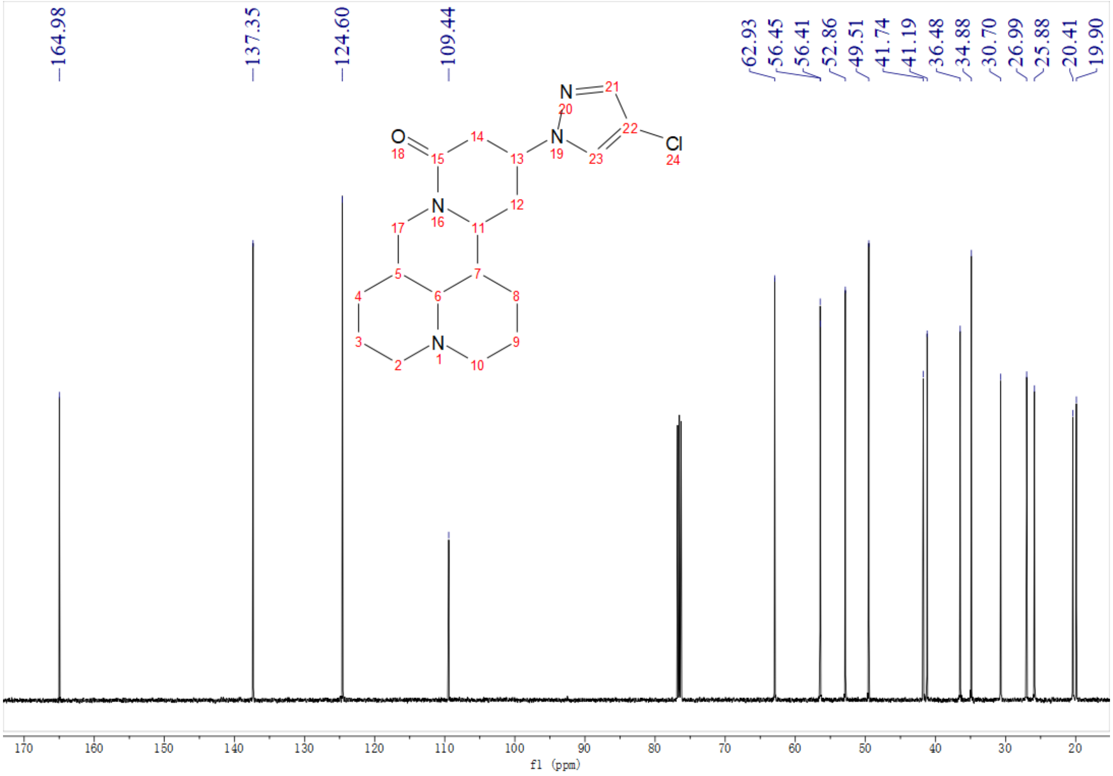

Figure S20 ^{13}C NMR spectrum of Compound 5

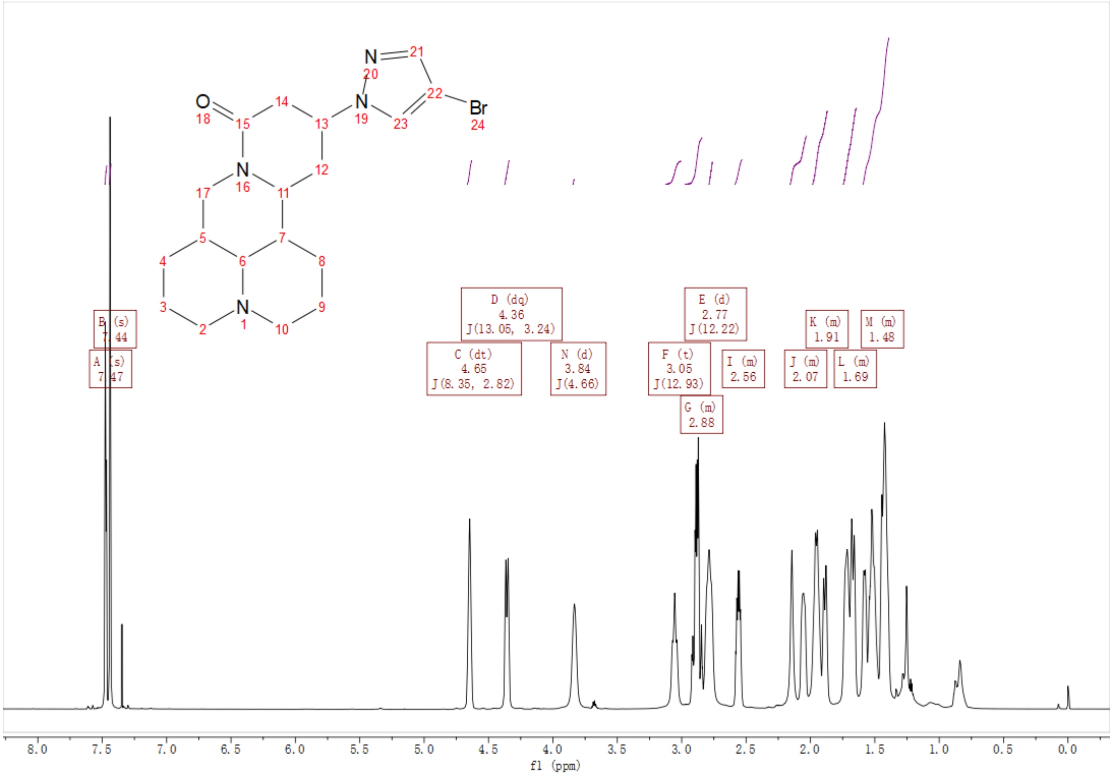

Figure S21 ^{1}H NMR spectrum of Compound 6

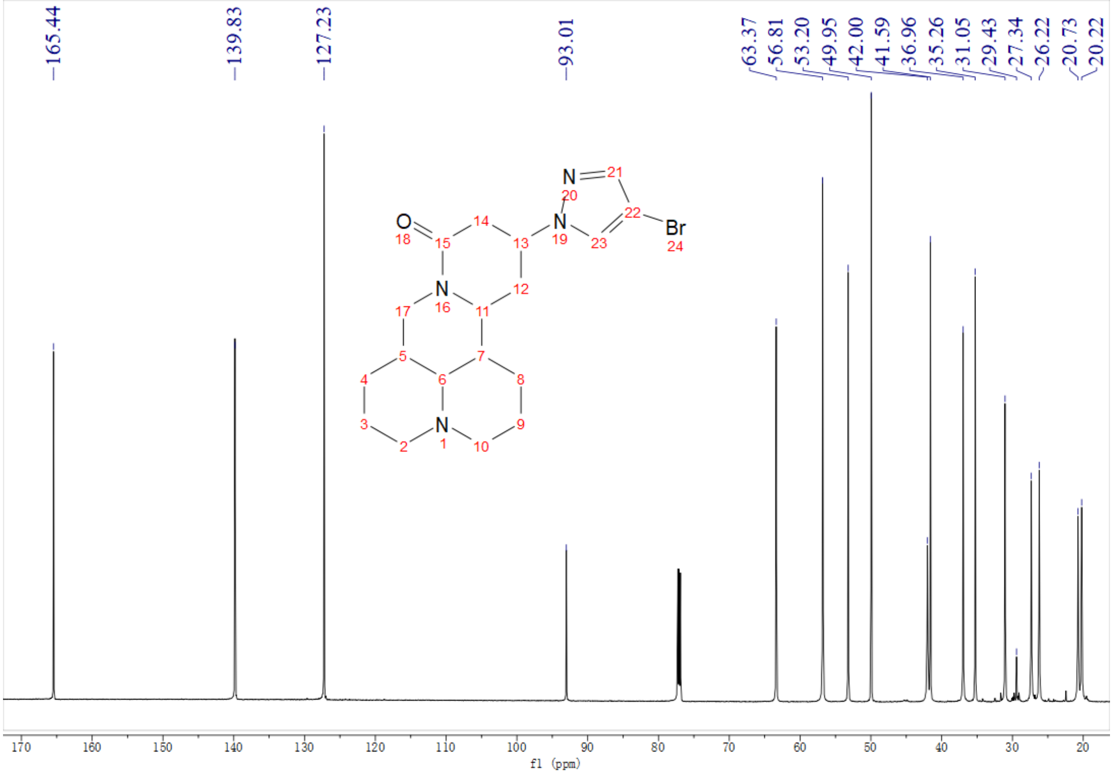

Figure S22 ^{13}C NMR spectrum of Compound 6

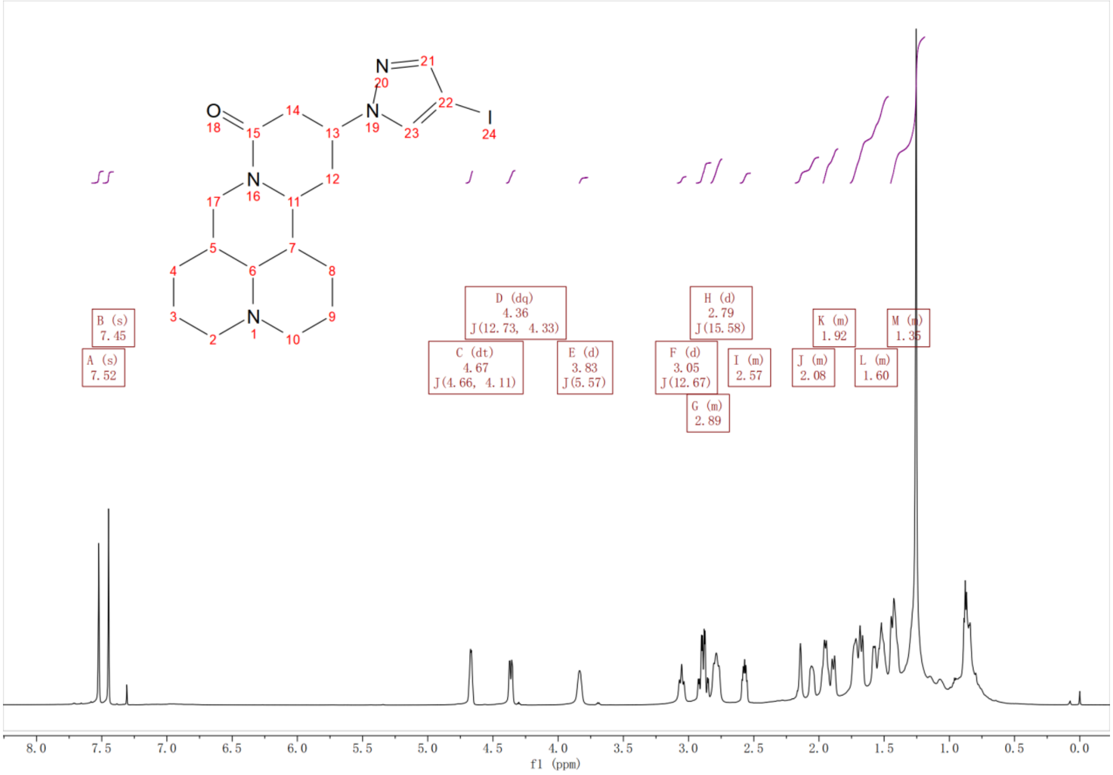

Figure S23 ^{1}H NMR spectrum of Compound 7

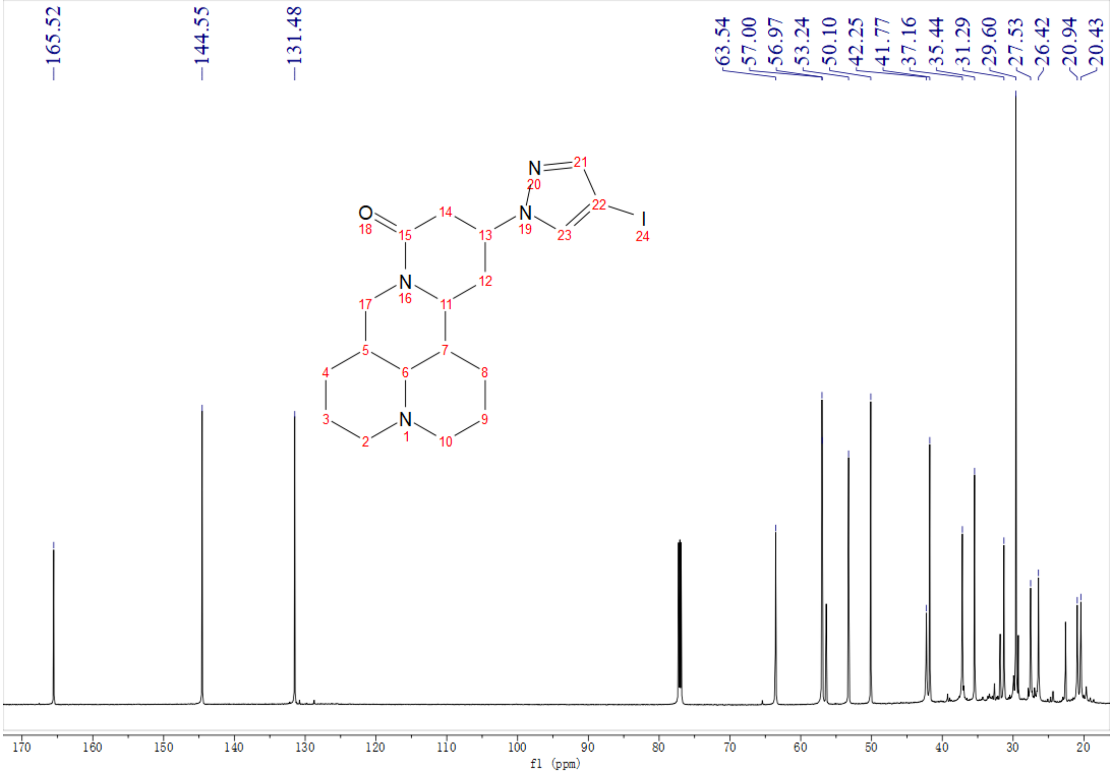

Figure S24 ^{13}C NMR spectrum of Compound 7

.X-ray structure of compounds 1~7

Table S1 Crystallographic data and details of refinement for compounds 1~7

	Compound 1	Compound 2	Compound 3	Compound 4	Compound 5	Compound 6	Compound 7
Empirical formula	$C_{18}H_{25}ClN_4O$	$C_{18}H_{25}BrN_4O$	$C_{18}H_{25}IN_4O$	$C_{18}H_{25}FN_4O$	$C_{18}H_{25}ClN_4O$	$C_{18}H_{25}BrN_4O$	$C_{18}H_{25}IN_4O$
Formula weight	348.88	393.33	440.33	332.42	348.87	393.33	440.33
Crystal system	monoclinic	orthorhombic	orthorhombic	orthorhombic	orthorhombic	orthorhombic	orthorhombic
Space group	$P2_1$	$P2_12_12_1$	$P2_12_12_1$	$P2_12_12_1$	P212121	$P2_12_12_1$	$P2_12_12_1$
a/Å	5.17160(10)	5.2191(3)	5.18980(10)	8.22130(10)	5.34760(10)	5.3364(4)	5.3090(3)
b/Å	12.1806(2)	27.5161(15)	12.3509(2)	8.27500(10)	14.4068(2)	14.5966(9)	14.9393(11)
c/Å	13.5833(2)	12.1953(6)	27.8491(4)	24.0526(3)	22.3285(4)	22.3188(16)	22.3783(12)
α/°	90	90	90	90	90	90	90
β/°	91.1060(10)	90	90	90	90	90	90
γ/°	90	90	90	90	90	90	90
Volume/Å^3	855.50(3)	1751.36(16)	1785.09(5)	1636.33(3)	1720.23(5)	1738.5(2)	1774.88(19)
ρ_{calc}g/cm^3	1.3543	1.492	1.6346	1.349	1.347	1.5027	1.6477
μ/mm^{-1}	2.074	3.296	14.193	0.765	2.063	3.32	14.275
F(000)	373.7	816	885.7	712	744	815.3	889.7
Independent reflections	3349<br>R_{int} = 0.0257<br>R_{sigma} = 0.0266	3676<br>Rint = 0.0386<br>Rsigma = 0.0329	3528<br>Rint = 0.0392<br>Rsigma = 0.0304	3213<br>Rint = 0.0304<br>Rsigma = 0.0374	3374<br>Rint = 0.0284<br>Rsigma = 0.0332	3627<br>Rint = 0.1132<br>Rsigma = 0.0838	3482<br>Rint = 0.0385<br>Rsigma = 0.0345
Goodness-of-fit on F^2	1.033	1.125	1.036	1.056	1.083	1.074	1.028
Final R indexes [I>=2σ (I)]	R_1 = 0.0289<br>wR_2 = 0.0775	R_1 = 0.0666<br>wR_2 = 0.2060	R_1 = 0.0353<br>wR_2 = 0.0965	R_1 = 0.0344<br>wR_2 = 0.0898	R_1 = 0.0329<br>wR_2 = 0.0826	R_1 = 0.0919<br>wR_2 = 0.2333	R_1 = 0.0453<br>wR_2 = 0.1213
Final R indexes [all data]	R_1 = 0.0299<br>wR_2 = 0.0794	R_1 = 0.0679<br>wR_2 = 0.2069	R_1 = 0.0358<br>wR_2 = 0.0968	R_1 = 0.0363<br>wR_2 = 0.0913	R_1 = 0.0351<br>wR_2 = 0.0838	R_1 = 0.0992<br>wR_2 = 0.2522	R_1 = 0.0468<br>wR_2 = 0.1237

Table S2 Geometric parameters of compounds 1-7 (Å, °)

	Compound 1	Compound 2	Compound 3	Compound 4	Compound 5	Compound 6	Compound 7
O18-C15	1.236(2)	1.238(9)	1.239(5)	1.237(2)	1.233(2)	1.242(8)	1.234(6)
N19-C13	1.460(2)	1.463(9)	1.461(5)	1.465(2)	1.468(2)	1.479(8)	1.462(7)
N20-C21	1.327(2)	1.312(10)	1.327(5)	1.340(3)	1.334(2)	1.325(9)	1.334(7)
C22-C23	1.372(2)	1.371(11)	1.369(6)	1.367(3)	1.371(3)	1.374(9)	1.385(7)
C21-X24/ C22-X24	1.7240(16)	1.884(7)	2.081(4)	1.348(2)	1.7210(19)	1.900(7)	2.076(5)
C14-C13-N19	109.27(13)	108.70(5)	108.8(3)	112.42(16)	108.99(13)	109.4(5)	109.70(4)
C23-N19-N20	112.44(13)	112.50(6)	112.30(3)	112.83(16)	112.58(15)	113.00(5)	112.40(4)
C21-N20-N19	103.34(13)	102.60(6)	103.50(4)	104.40(17)	104.76(15)	105.00(5)	105.00(5)
C23-C22-C21	103.85(14)	103.00(7)	103.50(4)	107.21(19)	105.93(16)	107.00(6)	106.60(5)
C22-C21-N20	113.28(14)	114.40(7)	113.30(4)	110.50(18)	110.72(17)	110.70(6)	110.70(5)

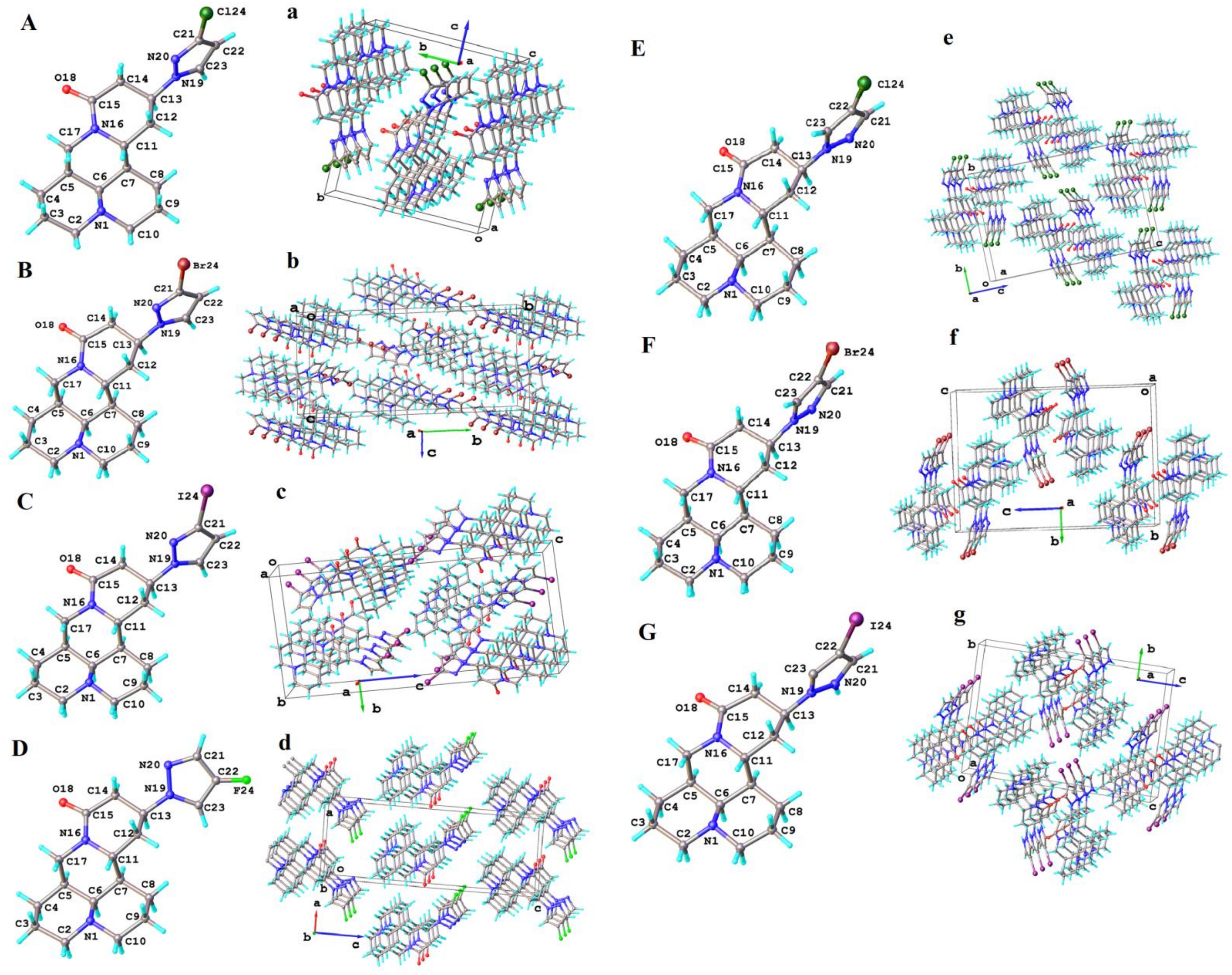

Figure S25 The crystal structure and packing picture of compounds 1-7

Degradability

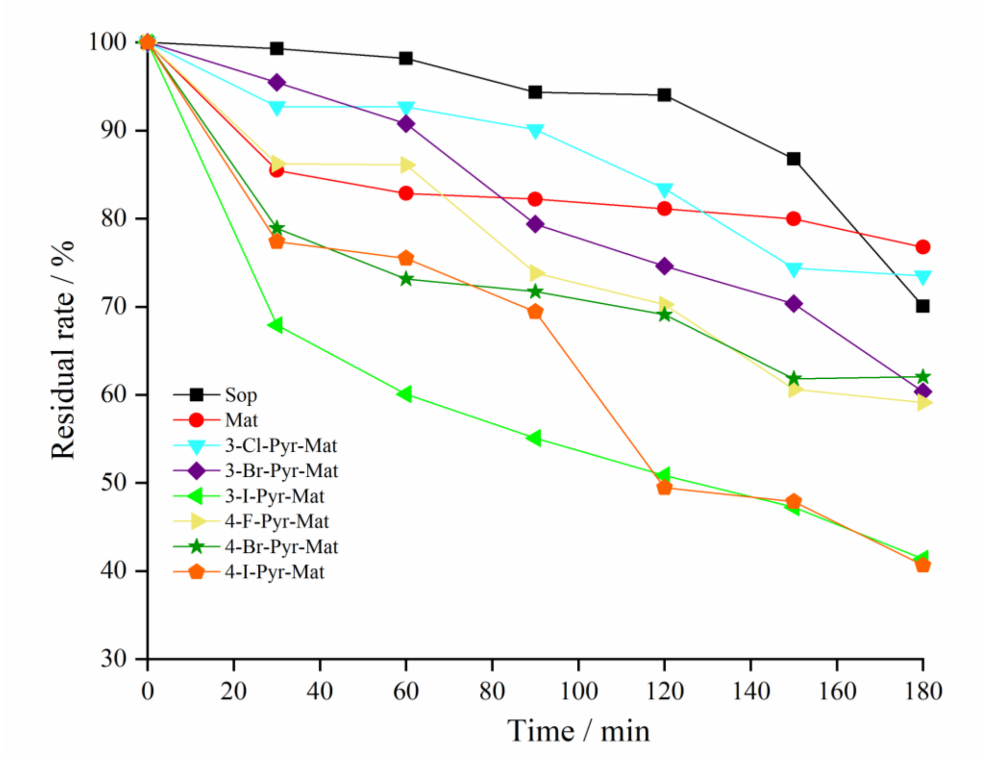

Figure S26 The photodegradation residual rate of matrine and its derivatives
